# Modulation of the Pharmacological Activities of Secretory Phospholipase A2 from *Crotalus durissus cascavella* Induced by Naringin

**DOI:** 10.3390/molecules16010738

**Published:** 2011-01-18

**Authors:** Marcelo L. Santos, Daniela O. Toyama, Simone C. B. Oliveira, Camila A. Cotrim, Eduardo B. S. Diz-Filho, Fábio H. R. Fagundes, Veronica C. G. Soares, Ricardo Aparicio, Marcos H. Toyama

**Affiliations:** 1 Laboratório de Biologia Estrutural e Cristalografia, Instituto de Química, UNICAMP, Campinas, São Paulo, Brazil; 2 Universidade Presbiteriana Mackenzie, CCBS, São Paulo, Brazil; 3 Departmento de Bioquímica, Instituto de Biologia, UNICAMP, Campinas, São Paulo, Brazil; 4 Laboratório de Macromoléculas Química, UNESP/CLP, São Vicente, São Paulo, Brazil

**Keywords:** secretoy phospholipase A2, *Crotalus durissus cascavella*, naringin, enzymatic activity pharmacological effects, small angle X-ray scattering

## Abstract

In this work we have characterized the action of the naringin, a flavonoid found in grapefruit and known for its various pharmacological effects, which include antioxidant, blood lipid lowering and anticancer activity, on the structure and biochemical activities of a secretory phospholipase A (sPLA2) from *Crotalus durissus cascavella*, an important protein involved in the releasinge of arachidonic acid in phospholipid membranes. sPLA2 was incubated with naringin (mol:mol) at 37 °C and a discrete reduction in the UV scanning signal and a modification of the circular dichroism spectra were observed after treatment with naringin, suggesting modifications of the secondary structure of the protein. This flavonoid was able to decrease enzymatic activity and some pharmacological effects, such as myonecrosis, platelet aggregation, and neurotoxic activity caused by sPLA2, however, the inflammatory effect was not affected by naringin. In addition, small angle X-ray scattering (SAXS) data were collected for sPLA2 and naringin-treated sPLA2 to evaluate possible modifications of the protein structure. These structural investigations have shown that sPLA2 is an elongated dimer in solution and after treatment with naringin a conformational change in the dimeric configuration was observed. Our results suggest that structural modification may be correlated with the loss of enzymatic activity and alterations in pharmacological properties.

## 1. Introduction 

Polyphenolic compounds, such as flavonoids and coumarins, are widely distributed in the plant kingdom. Some of these compounds have interesting medicinal properties, exerting anti-lipoperoxidant, anti-inflammatory, anti-allergic, antiviral, antibacterial, and anticancer effects [1]. Recent studies have shown that flavonoids from various plants induce an *in vitro* dose-dependent inhibition of phospholipid hydrolysis mediated by both secretory and cytosolic phospholipasesA2 (sPLA2 and cPLA2, respectively) [2,3]. Flavonoids assayed *in vivo* have also shown a significant anti-inflammatory effect against acute edema induced by carrageenan [4].

It has been shown that flavonoids inhibit group I sPLA2s from porcine pancreas and *Naja naja* in a different way from group II sPLA2 from *Vipera russelii* and *Crotalus atrox*. The most important regions involved in the inhibition of sPLA2 were reported to be hydroxyl groups in the 30- and 40-positions [5,6]. In addition, Iglesias *et al.* [7] showed that flavonoids such as morin modify the secondary structure of snake venom sPLA2 similarly to sPLA2 when modified with 7-hydroxy coumarin [8]. Group II sPLA2 enzymes have been found at inflammatory sites in animal models, as well as in synovial fluid from patients with rheumatoid arthritis and various human inflammatory diseases in which a correlation between serum sPLA2 levels and disease activity is observed [9,10]. Moreover, exogenous administration of sPLA2, such as snake venom sPLA2, induces or exacerbates the inflammatory response in animals [11,12]. Structural analyses have revealed that snake venom sPLA2s have a similar molecular profile as human secretory PLA2s as well as a conserved catalytic site [13].

Naringin, hesperidin, and neohesperidin have been described as some of the most abundant flavanones found in the *Citrus* genus, and are responsible for many of the pharmacological properties of *Citrus* flavonoids. These flavanones are particularly interesting because of their antitumoral and anti-inflammatory effects, which are linked to the abilities of these compounds to inhibit enzymes involved in cell activation [14]. Because sPLA2 from snake venom has been characterized as a pro-inflammatory inducer, we investigated for the first time the effect of naringin on the edematogenic activity of sPLA2 from *Crotalus durissus cascavella* venom and evaluated other biological and pharmacological activities induced by this protein.

In addition, we also investigated the structural modification induced by this polyphenolic compound on the structure of sPLA2. Like other flavonoids, naringin displays a combination of multiple hydroxyl groups substituting a ketone-containing flavonoid skeleton and a glycoside group [15]. Reports on flavonoid structure−function relationships have demonstrated that antioxidant activities and enzyme inhibition are dependent on particular flavonoid structural motifs, such as structure oxidation levels, substituents (position, number, and nature of groups), and the presence of glycosylation [14]. Phenolics compounds, particularly flavonoids, have been shown to possess important antioxidant activity toward free radicals, which are associated with the natural metabolism of aerobic cells [16].

To obtain structural information from native sPLA2 and sPLA2 after naringin treatment (sPLA2-Nar), we used a variety of solution-based techniques. Because small angle X-ray scattering (SAXS) is a sensitive technique for analyzing global shape and dimensions of macromolecules in solution [17], we employed this method together with molecular modeling, circular dichroism, and bioinformatic analysis.

Several sPLA2 crystallographic studies have been reported describing the main structural features of this class of proteins [18] and the correlations with biochemical properties [19]. However, only a few reports have been published regarding SAXS analysis of PLA2 [20,21]. In these studies, two different oligomeric states were observed for sPLA2s in solution: monomeric for sPLA2 from *Cerrophidion godmani* [20] and dimeric for sPLA2 from *Bothrops jararacussu* [21].

In addition, electrophoresis and spectroscopic studies have suggested that the dimer-like structure in solution is common to sPLA2s from other snake venoms [22,23]. Two main different conformations were reported using crystallographic models for these dimers: an extended conformation maintained by polar interactions formed between the N-terminal helix regions and β-wing residues of each monomer, and a compact dimeric organization of the molecules in which the hydrophobic channels are buried [24,25]. In solution, however, only the compact conformation has been observed [21]. In this paper, we describe a new extended dimer configuration for PLA2 in solution. The low resolution model obtained for sPLA2-Nar presents an interesting bending in comparison with the envelopes for the native sPLA2 that seem to be correlated with the enzymatic and pharmacological alterations observed in our studies.

## 2. Results and Discussion 

**Figure 1:**
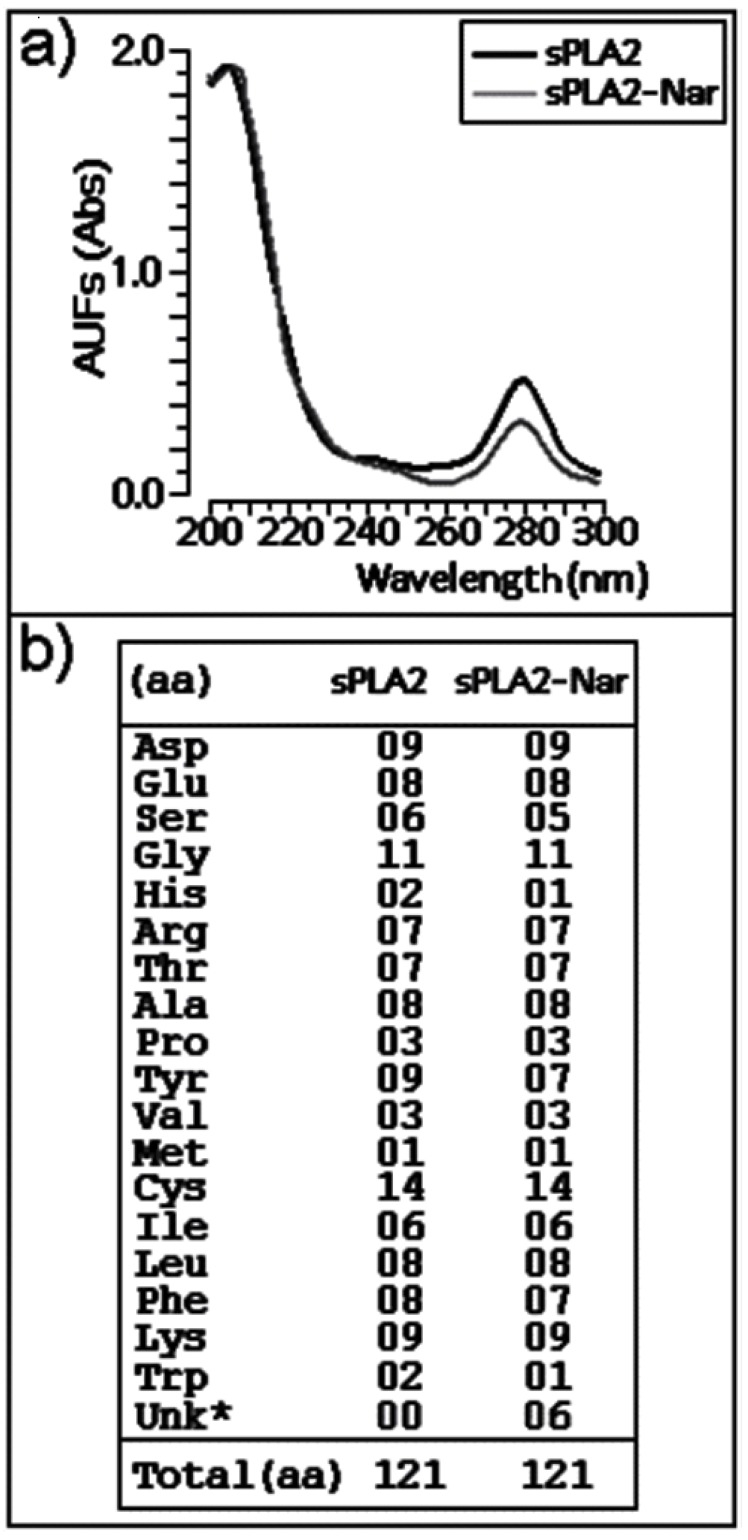
**(a)** Absorption spectra of sPLA2 and modified sPLA2 after incubation with naringin at wavelength intervals of 200–300 nm. The amino acid modification was analyzed at 280 nm. **(b)** Amino acid composition of native sPLA2 and sPLA2-Nar. UNK indicates unknown or unidentified peak or extra peaks, identified by the PICO-TAG amino acid analysis system.

Chemical treatment of purified sPLA2 with naringin was conducted as described by Iglesias *et al.* [7]. Under these conditions, a discrete decrease in the absorbance of sPLA2-Nar in the wavelength region between 270–285 nm (Figure 1a) was observed, which can be attributed to the spectroscopic absorption of the aromatic amino acid residues. Amino acid analysis indicated that naringin mainly induced modification on the aromatic amino acid residues and histidines, similar to what was reported in a previous study with morin [7]. However, we observed slight changes in serine composition (Figure 1b).

**Figure 2:**
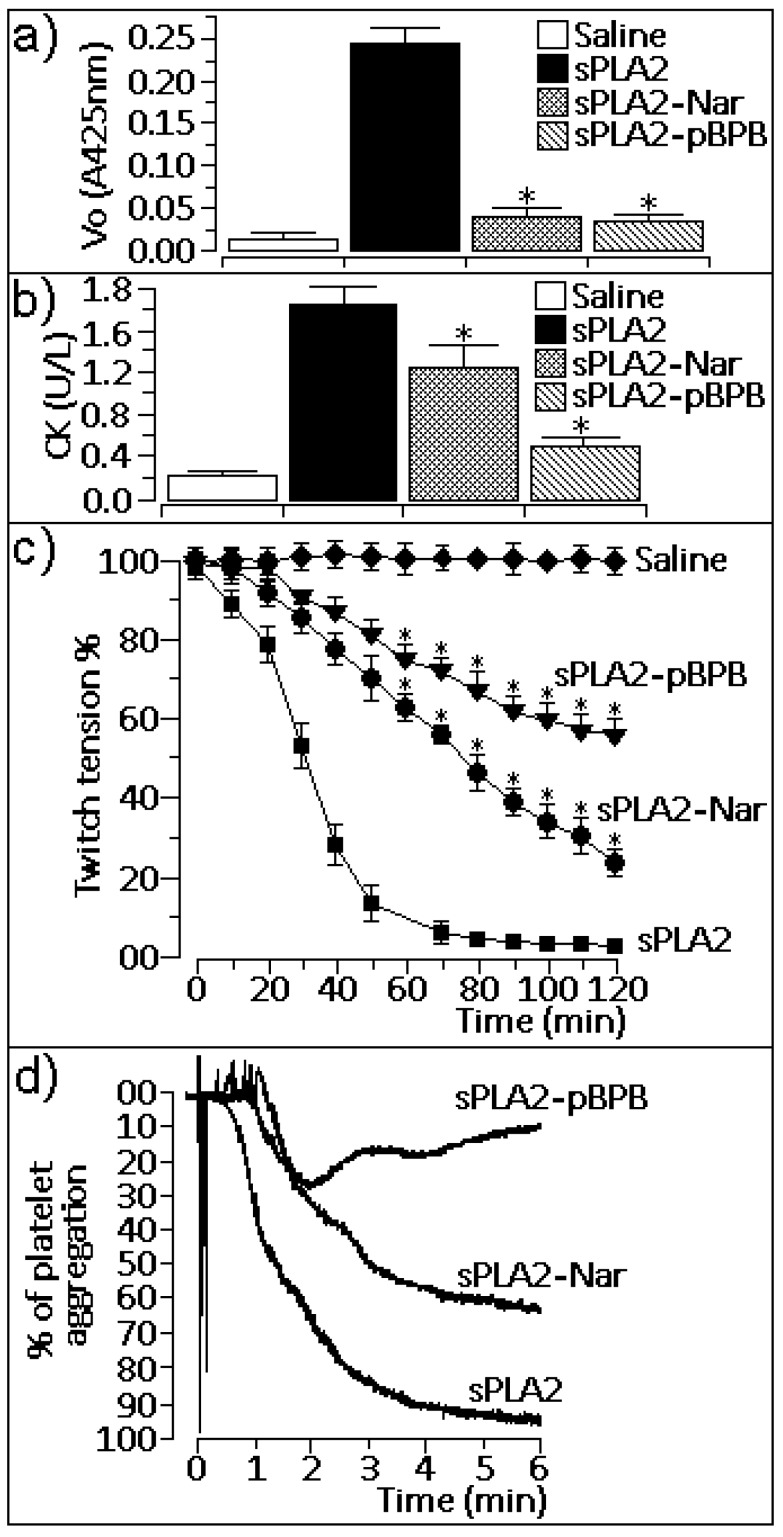
**(a)** Enzymatic activity. sPLA2-Nar showed a significant decrease compared to native sPLA2 and was similar to sPLA2 treated with 4-bromophenacyl bromide (sPLA2-p-BPB) (n = 12, *P < 0.05). **(b)** Measurement of creatine kinase (CK) activity from samples of animals treated with saline (negative control), native sPLA2 (50 μL, 50 μg), or (sPLA2-Nar; 50 μL, 50 μg). The last bar represents the effect of sPLA2 treated with p-BPB (50 μL, 50 μg). Results of creatine kinase levels were expressed as units of enzymatic activity per liter (U/L) of blood. Each column represents six animals, and error bars indicate the S.E.M. *P < 0.05 compared with sPLA2 native. **(c)** Neurotoxic effect of sPLA2 and sPLA2-Nar on the chick biventer cervicis preparation. Results were expressed as a percent modification of twitch tension, and each point represents the mean ± S.E.M. of five preparations. * P < 0.05. **(d)** The washed platelets assay was performed with venous blood collected from healthy volunteers. The blood was centrifuged in 3.8% trisodium-citrate for 10 minutes at 200 g, and the residue was centrifuged again for 20 minutes at 1,500 g. Aggregations were performed using native sPLA2 and sPLA2-Nar.

The enzymatic activity of native sPLA2 was virtually abolished by treatment with naringin, which showed similar inhibition with p-BPB (Figure 2a; n = 12 experiments; p < 0.05). Native sPLA2 induced a moderate myotoxic effect (Figure 2b). Previous treatment of sPLA2 with naringin reduced the myotoxic effect induced by sPLA2 2-fold, whereas *p*-bromophenacyl bromide (p-BPB) abolished this effect (Figure 2b; n = 6 experiments; P < 0.05). Native sPLA2 induced irreversible neuromuscular blockage 70 minutes after injection of the toxin into chick *biventer cervicis* muscle (Figure 2c), whereas sPLA2 treated with naringin induced a moderate blockage when compared to native sPLA2. Although both naringin and p-BPB virtually abolished the enzymatic activity of sPLA2, only p-BPB treatment irreversibly abolished the neurotoxic effect induced by sPLA2 (Figure 2c; n = 6 experiments). Platelet aggregation induced by native sPLA2 was strongly reduced by treatment with naringin and was abolished by treatment with p-BPB (Figure 2d, n = 4 experiments for each drug).

**Figure 3:**
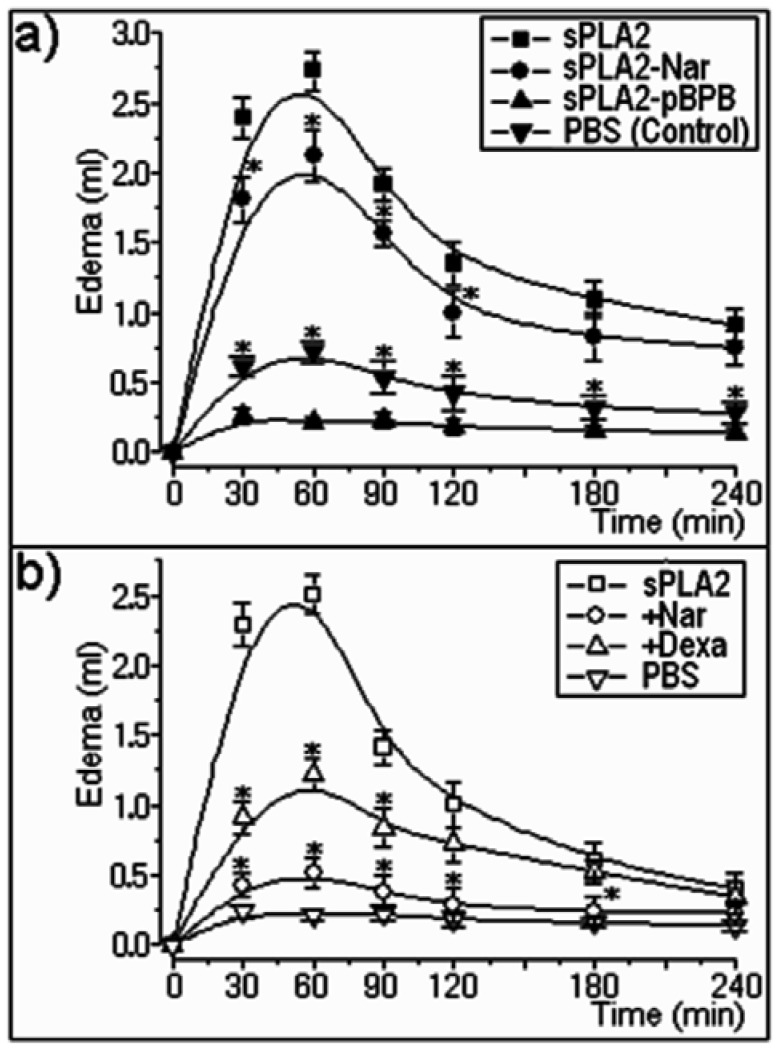
**(a)** Rat paw edema induced by sPLA2 and sPLA2-Nar showed dose-dependent edema with doses of 10 μg/paw, whereas p-BPB treatment virtually abolished the edema induced by sPLA2. Observations were conducted at times of 30, 60, 90, 120, 180, and 240 minutes after injection. Results were expressed as the increase in paw volume (mL) calculated by subtracting the basal volume. Each point represents the mean ± S.E.M. of four rats and *P < 0.05. **(b)** Effect of previous injection of dexamethasone or naringin (3.0 mg/kg) before injection of native sPLA2. The results were expressed as microliters of plasma protein extravasation. Each point represents the mean of four animals, and each error bar indicates the S.E.M. *p < 0.05 compared with sPLA2 control.

Subplantar injections of treated and non-treated sPLA2 (5 µg/paw; n = 5) induced significant acute edema (p < 0.05) when compared to the control (PBS) (Figure 3a). Naringin treatment induced a slight decrease in edema volume in comparison to native sPLA2, whereas p-BPB strongly reduced this effect. On the other hand, previous injection of naringin into the paw affected the edema formation. In this new assay, sPLA2-Nar prevented the edema induced by native sPLA2, whereas dexamethasone (Dexa), a potent synthetic anti-inflammatory compound, significantly reduced this pharmacological effect (Figure 3b).

**Figure 4:**
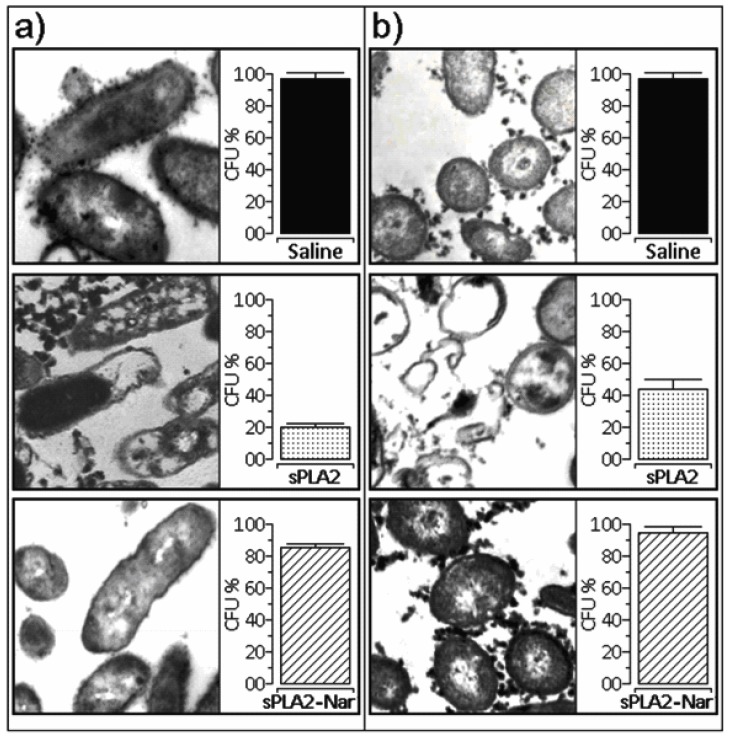
**(a)** Effect of naringin on the sPLA2 antibacterial activity against *Staphylococcus mutans*. b) Observed structural modifications induced by sPLA2 and sPLA2-Nar on *Xanthomonas axonopodis pv passiflorae*. Saline was used as a control. CFU: Colony Forming Units.

Native sPLA2 showed antibacterial activity against *Xanthomonas axonopodis pv passiflora* and *Streptococcus mutans*. In both cases, we observed that sPLA2 induced a strong modification of the bacterial cell membrane (Figure 4a and 4b). These results suggest that the enzymatic activity of sPLA2 is important for antibacterial activity, and that inhibition of sPLA2 enzymatic activity by naringin virtually abolished its antibacterial activity (Figure 4a and 4b).

**Figure 5:**
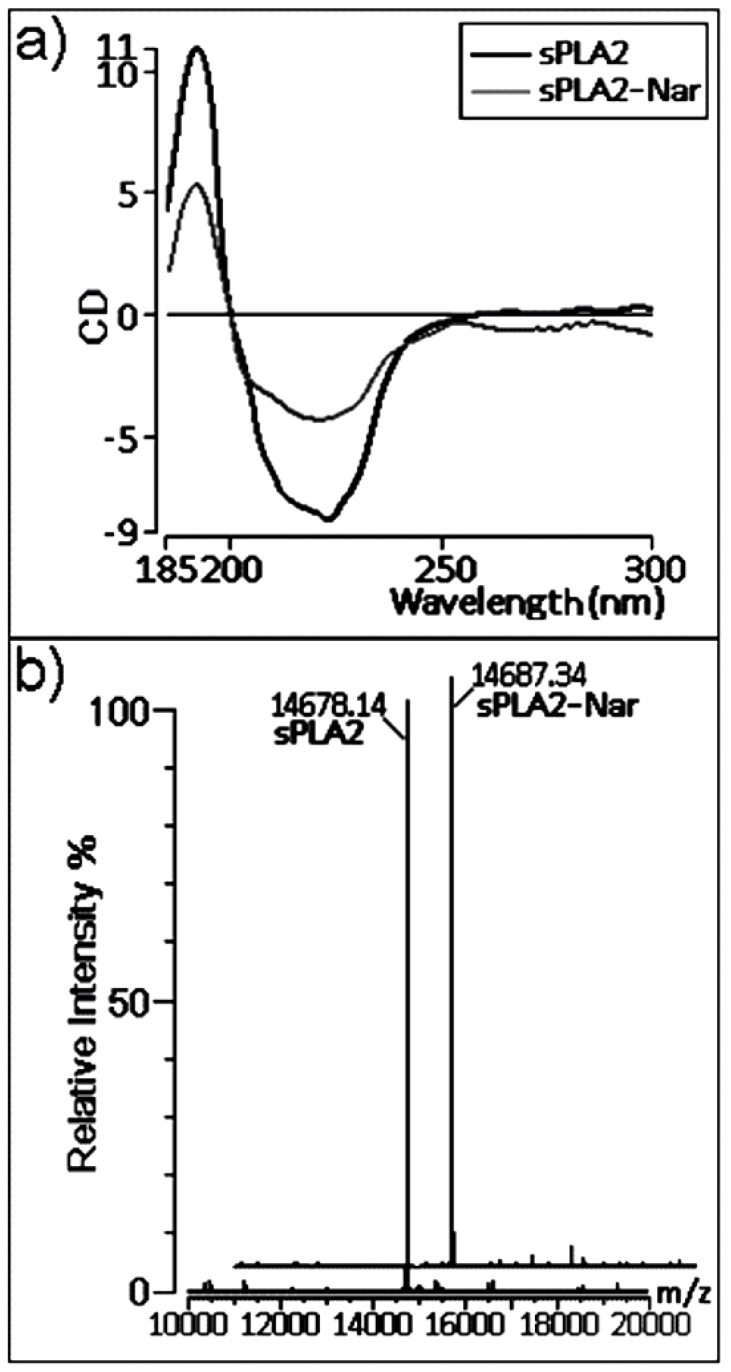
**(a)** CD spectra of native sPLA2 and sPLA2-Nar. Data over the range of 185–300 nm are shown. The CD spectra are expressed in ellipticity units in millidegrees. **(b)** The mass measurement of native and naringin-treated sPLA2 by MALDI-TOFF mass spectrometry resulting in a molecular mass of 14,678.14 Da for native sPLA2 and 14,687.34 Da for sPLA2-Nar.

We also performed chemical analyses to better understand the effects observed on pharmacological activities. Circular dichroism (CD) measurements of native sPLA2 and sPLA2-Nar showed a significant decrease in the CD signal due to treatment with the flavonoid (Figure 5a). This spectroscopic alteration suggests that the residues modified by naringin are important secondary structural elements.

Mass spectrometry showed that sPLA2 had an increase in its mass-to-charge ratio after naringin treatment in comparison with native sPLA2 (Figure 5b). Similar to the alterations observed in the CD spectra, the increase in the molecular mass of sPLA2 is a consequence of the chemical modification induced by naringin.

The effect of naringin treatment on sPLA2 from *C. d. cascavella* was evaluated with a systematic structural study. Despite several attempts to crystallize this protein, it was not possible to obtain a high resolution model through X-ray crystallography. Therefore, we employed an alternative strategy to acquire useful structural information to interpret the biochemical and pharmacological results obtained.

Initially, we performed a homology modeling calculation and secondary structure analysis using SWISS-MODEL and PSIPRED web servers, respectively. The *C. d. cascavella* sPLA2 homology model was built based on the template structure of crotoxin B from *Crotalus durissus terrificus* snake venom (PDB ID: 2QOG, chain C) [26], which shows 90% sequence identity. According to secondary structure prediction, the model of sPLA2 from *C. d. cascavella* showed the same structural motifs of PLA2: a β-wing, a hydrophobic channel, a calcium-binding loop, and C-terminal regions.

**Figure 6:**
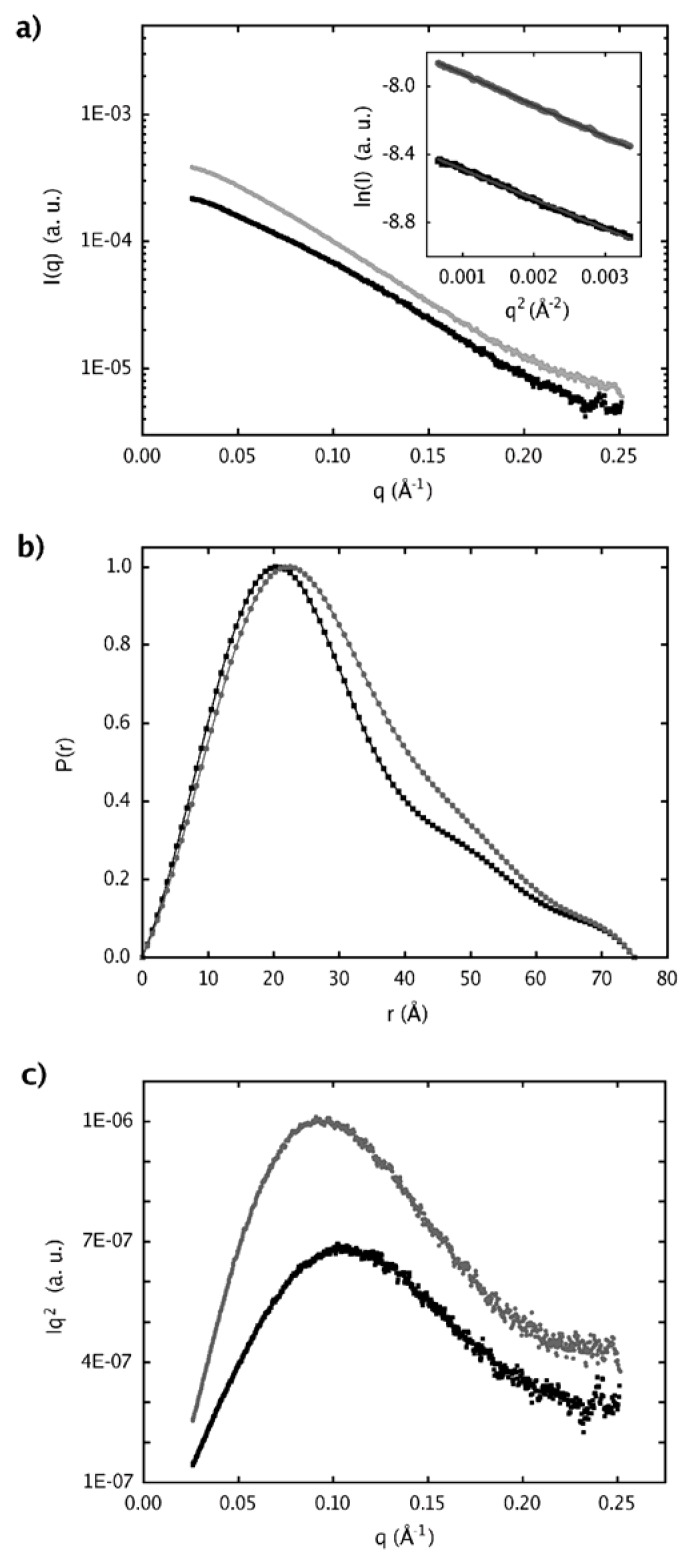
Small Angle X-ray Scattering data plots. **(a)** Experimental scattering curves and Guinier region with its linear data fit. **(b)** Distance distribution functions, p(r). **(c)** Kratky plot. Black squares represent the SAXS data for the native protein (sPLA2, ~4 mg/mL), and the SAXS data for the protein after treatment with naringin (sPLA2-Nar, ~7 mg/mL) are shown as gray circles.

Structural information was also obtained using SAXS in which tertiary and quaternary structures of native sPLA2 and sPLA2-Nar were investigated. The scattering curves for both native and treated sPLA2 with the associated distance distribution functions, and Guinier and Kratky plots are shown in Figure 6. The Guinier plot shown in the inset of Figure 6a exhibits a linear relationship within the interval of 0.026 Å-1 < q < 0.058 Å-1 for sPLA2 and 0.026 Å-1 < q < 0.056 Å-1 for sPLA2-Nar, which are characteristic of monodisperse systems and from which the radius of gyration (Rg) of 23 Å and 24 Å were derived, respectively. 

The p(r) functions calculated with GNOM from the scattering curves are shown in Figure 6b. For both native sPLA2 and sPLA2-Nar, the Kratky plot exhibited a characteristic maximum of correctly enovelated proteins (Figure 6c). The radii of rotation calculated for several sPLA2 crystallographic models with different numbers of molecules in the asymmetric unit (ASU) (PDB ID: 1A2A, 1BJJ, 2QOG, 1CL5, 1JIA, and 2OQD) using the program CRYSOL led to a regular dependency of Rg values with the number of molecules in the ASU: ~35 Å for eight, ~30 Å for six, ~25 Å for four, from 18 to 24 Å for two, and from 13 to 14 Å for one molecule, suggesting that sPLA2 from *C. d. cascavella* in solution, before and after treatment with naringin, is composed of two polypeptide chains.

**Figure 7:**
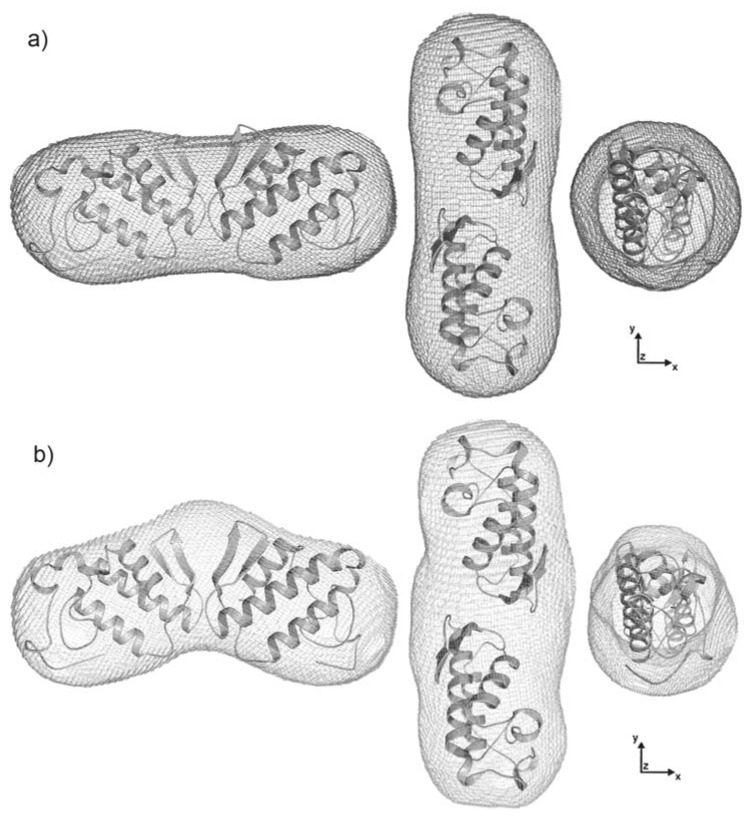
Superimposition of the dimeric high resolution model for agkistrodotoxin sPLA2 (PDB entry 1BJJ, chains A and E) is shown as a cartoon, and SAXS envelopes are shown in dark gray and light gray. **(a)** The native low resolution model obtained is shown in dark gray. **(b)** The envelope of the protein after treatment with naringin is shown in light gray. Envelopes (left) are rotated clockwise by 90° around the y- (middle) and z-axes (right). This figure was prepared using PyMOL (DeLano Scientific, San Carlos, CA, http://www.pymol.org).

Determination of the molecular weight with the web tool “SAXS MoW” confirmed that sPLA2 and sPLA2-Nar appear as dimers in solution at the concentrations evaluated, with an average molecular weight of ~28 kDa. The expected molecular weight based on the amino acid sequence of sPLA2 is 28.9 kDa. Low resolution models recovered from scattering data showed an elongated shape as expected by the distance distribution functions asymmetric format (Figure 6b). In addition, envelope models of sPLA2-Nar were interestingly different from the native envelopes (Figure 7).

According to our models, naringin appears to induce a conformational modification of sPLA2 from *C. d. cascavella*. To interpret such envelopes, different dimer configurations of several sPLA2 crystallographic models found in PDB were superimposed, including dimers formed by symmetric mates (results not shown). Using the program CRYSOL to compare the experimental scattering data and the scattering intensities computed from these models, the lowest discrepancy was found for the dimer made of chains A and E of agkistrodotoxin from *Agkistrodon halys pallas* venom (PDB ID 1BJJ) [27]. Analysis of protein assemblies and interfaces using the PISA web server suggested that assembly of chains A and E of agkistrodotoxin is stable in solution, showing one of the highest ΔdissG, equal to 6.7 kcal/mol.

**Figure 8:**
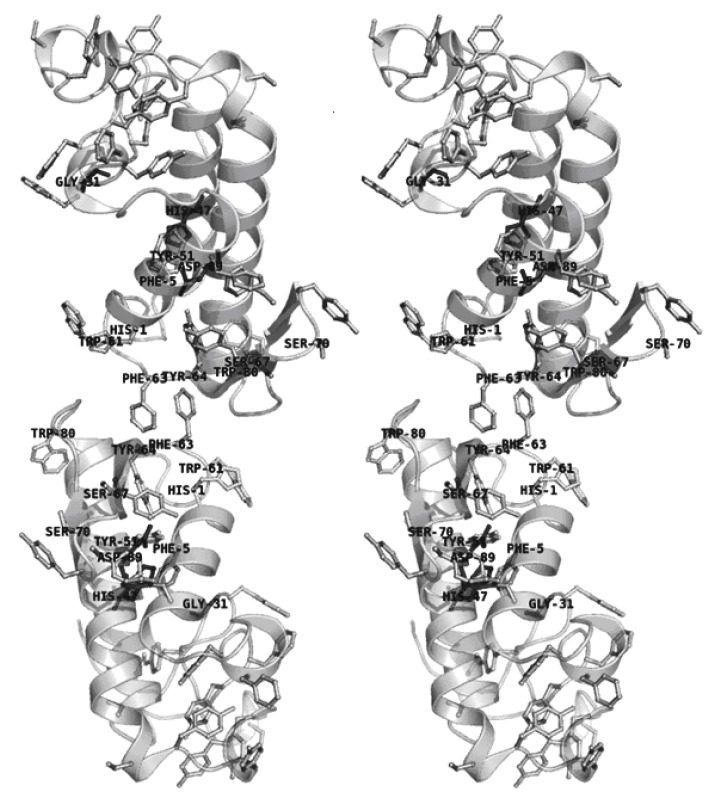
Stereo cartoon representation of the *C. d. cascavella* sPLA2 dimer modeled with SWISS-MODEL and built *in silico* based on the high resolution model of agkistrodotoxin sPLA2 (PDB entry 1BJJ, chains A and E. This figure was prepared using PyMOL (DeLano Scientific, San Carlos, CA, http://www.pymol.org).

Finally, the *C. d. cascavella* sPLA2 homology model obtained using the SWISS-MODEL web server was superposed onto chains A and E of the agkistrodotoxin dimer in an attempt to evaluate biochemical and structural alterations in sPLA2 after naringin treatment (Figure 8). This new dimeric assembly built in silico for *C. d. cascavella* sPLA2 was then used in the interpretation of the low resolution model bending due to naringin treatment.

### 2.1. Enzymatic activity 

Polyphenolic compounds, such as naringin, have been characterized as highly specific and potent inhibitors of phospholipase A2 activity. This action of flavonoids and other polyphenolic compounds is attributed to an ability to bind the hydrophobic catalytic pocket of this enzyme, such as is found in morin, and umbelliferone incubated with sPLA2 [7,8]. In addition, it has been reported that the positions of the hydroxyl groups found in the flavonoid structure (3′- and 4′-position in the B-ring) are important for selective inhibition of sPLA2 activity of group II molecules, such as PLA2s from *Crotalus durissus ssp* and *Bothrops sp* [5].

According to our results, the enzymatic activity of native sPLA2 from *C. d. cascavella* was strongly reduced after treatment with naringin, almost to the same level as the potent sPLA2 enzymatic inhibitor p-BPB (Figure 2a). These findings suggest that naringin and p-BPB may inhibit sPLA2 activity in a different way. Crystallographic studies of sPLA2 from *Bothrops jararacussu* venom have shown that pBPB interacting with His-48 an important residue involved with the the catalytic triad, whereas our results suggest that naringin modified several aromatic residues in the hydrophobic channel [28]. 

### 2.2. Pharmacological properties

In addition to the enhanced decrease in catalytic activity, sPLA2 inhibitors recognize pharmacological sites of such enzymes, consequently affecting their pharmacological action [29]. As our studies showed, chemical treatment of sPLA2 with naringin reduced some pharmacological effects, including myotoxicity (Figure 2b), neurotoxicity (Figure 2c), pro-platelet aggregation activity (Figure 2d), and edema formation (Figure 3a), which were induced by native sPLA2 in comparison with the same assays performed with the inhibitor p-BPB.

One possible explanation for the lack of association between pharmacological and enzymatic activities is that the chemical modification of some amino acids induced by naringin, in particular aromatic amino acids and histidines, affected the toxin’s ability to interact with the pharmacological receptor, but did not lead to abolishment of this function. Our results and those described by Cardoso *et al.* [30] demonstrate that enzymatic activity of sPLA2 is not crucial for pharmacological activities of this sPLA2 isolated from *C. d. cascavella* venom.

In terms of edema formation, it is noteworthy that the pharmacological effect seems to not be correlated with the catalytic activity, and that when naringin was previously applied to animal hindpaws, strong inhibition of hind paw edema was seen (Figure 3b). Flavonoids and other organic compounds from plants, such as flavones and alkaloids, are known to inhibit the enzymatic activity of sPLA2 [2,3], as well as lipoxygenase/cyclooxygenase and citossolic PLA2, enzymes evolved in the inflammatory cascade [31]. We believe that sPLA2s are responsible for activating these inflammatory enzymes due to arachidonic acid release and initiation of the inflammatory process. Thus, when naringin is previously injected, it inhibits these enzymes and prevents edema formation.

This complementary assay suggests that unlike other pharmacological properties, the edema induced by sPLA2 is dependent on membrane phospholipids hydrolysis generating pharmacologically active lipids. On the other hand, the pharmacological modes of action of sPLA2 on myotoxicity, neurotoxicity, and platelet aggregation are not completely related to the enzymatic activity but may be involved in the ability of specific sPLA2 molecular regions to recognize pharmacological sites on the cells, as proposed by Cardoso *et al.* [30] for crotoxin, a PLA2 from *Crotalus durissus terrificus* venom.

The antibacterial property induced by native sPLA2 from *C. d. cascavella* appears to be a toxic effect that is more closely related to the enzymatic activity, as was found for other sPLA2 that employ enzymatic catalysis for antibacterial function [32]. Although native sPLA2 showed high phospholipase function, bacterial cell membrane damage was significant. After naringin treatment and pronounced reduction of catalytic activity, the level of lysis was also decreased (Figure 4). Thus, abolishing or altering the sPLA2 enzymatic apparatus led to a significant loss of antibacterial activity by sPLA2 from *C. d. cascavella* venom.

### 2.3. Chemical and structural modifications

The observed UV absorption modification after sPLA2 treatment with naringin can be mainly attributed to chemical changes in the aromatic amino acid residues and histidines of this enzyme, as verified previously by Iglesias *et al.* [7]. In addition, mass spectrometry analysis demonstrated that the chemical shift induced by naringin was discrete, not indicating an attachment of naringin to the sPLA2 structure. The same result was found in previous studies of sPLA2 treated with umbelliferone [8].

CD spectroscopy showed that naringin treatment induced considerable alteration in the CD signal of sPLA2 as a consequence of modifications of mainly the α-helix. Helical structures in sPLA2 are responsible for forming the hydrophobic channel [18], which is occupied by the acyl chains of the substrate when bound, positioning the cleavage center towards the catalytic residues and forming an aperture to the external surface. In class I and II sPLA2s, when no substrate is bound, the hydrophobic channel is filled with ordered water molecules [33]. Therefore, in addition to our spectroscopic analyses, structural characterization reports suggest that naringin predominantly acted in the hydrophobic channel of sPLA2 from *C. d. cascavella*.

Although side-chain orientation is still a main challenge in determining protein structures from a known sequence [34], methods based on rotamer libraries [35] have improved the prediction reliability in terms of side-chain torsional angles for preferred conformations [34]. However, amino acid side-chains in both crystallographic and molecular modeling models are known to differ from those in physiological conditions [36]. From this point of view and considering the results derived from our current work, the definition of the molecular region where naringin acts is more important than addressing specific interactions and the most probable biologically active side-chain conformations of the important amino acids.

### 2.4. Dimer models in solution for sPLA2 from C. d. cascavella 

Structural analyses of native sPLA2 and sPLA2-Nar performed by SAXS indicated that both samples behave as dimers in solution. Using high sequence identity snake venom sPLA2 crystallographic models to interpret our low resolution models, we observed that *C. d. cascavella* dimers presented a new extended configuration that has never been seen for PLA2 in solution.

We also verified that treatment with naringin induced conformational changes in sPLA2 with a clear bending of the dimer. Although unsuccessful, several attempts to model this bent dimer were performed, employing rigid body modeling and flexible docking. However, the interpretation of the SAXS model based on the *C. d. cascavella* homology model superimposed on the agkistrodotoxin crystallographic “dimer” (PDBID: 1BJJ, chains A and E) enabled identification of the aromatic amino acids, histidine and serine, of *C. d. cascavella* that are present at the dimer interface (Figure 8). These interfacial residues correspond to those affected by the action of naringin, and thus, the chemical modification caused by naringin must be responsible for the conformational change observed in the dimer configuration after naringin treatment.

It has been reported that the dimerization of snake venom sPLA2 is a common event that strongly increases the enzymatic activity of sPLA2 in solution and determines the allosteric behavior of PLA2 from *Crotalus durissus ssp* and *Bothrops sp* [13,37,38,39,40]. Our low resolution models for sPLA2 and sPLA-Nar allow us to suggest that in addition to chemical, secondary, and tertiary modifications induced by naringin in sPLA2 from *C. d. cascavella*, dimer bending may have reduced the possible enzymatic allosteric effect presented by this protein.

## 3. Experimental

### 3.1. Venom, animals and reagents

Venom from *C. d. cascavella* was kindly donated by the Institute of Butantan (São Paulo, Brazil). Naringin was obtained from Sigma Co., Ltd. (USA). Solvents, chemicals, and reagents used in protein purification and characterization were of HPLC grade or higher and were acquired from Sigma-Aldrich chemicals, Merck (USA), and Bio-Rad (USA). Male and female Wistar rats (120–150 g) and Swiss mice (18–20 g) used in the pharmacological assays were obtained from the Multidisciplinary Center of Biological Investigations (CEMIB-UNICAMP). All animal experiments were approved by the State University of Campinas Ethics Committee (São Paulo, Brazil).

### 3.2. Purification of sPLA2 from Crotalus durissus cascavella venom 

Whole venom was first fractionated as previously described by Oliveira *et al.* [41]. Dried venom (45 mg) was dissolved in ammonium bicarbonate buffer (0.2 M, pH 8.0) and clarified by centrifugation (4,500 g, 1 min). The supernatant was injected onto a molecular exclusion HPLC column (Superdex 75, 1 × 60 cm, Pharmacia), and the chromatographic run was developed with a flow rate for fraction elution of 0.2 mL/min. Absorbance was monitored at 280 nm. The separated crotoxin-like fraction was immediately lyophilized. The crotoxin-like fraction was then subjected to reverse phase chromatography using a μ-Bondapack C18 column (0.39 × 30 cm) with a flow rate for fraction elution of 1 mL/min. The chromatography was monitored at 280 nm. sPLA2 from *C. d. cascavella* was eluted using a non-linear gradient with buffer A (0.1% of trifluoroacetic acid in Milli-Q water) and buffer B (acetonitrile 66% in buffer A). The resulting PLA2 was termed sPLA2, and its purity was evaluated by Tricine SDS-PAGE and mass spectrometry on a MALDI-TOF mass spectrometer, as previously described by Toyama *et al.* [37,42].

### 3.3. Incubation of sPLA2 with naringin and purification of modified sPLA2

The incubation of sPLA2 with naringin (mol:mol) was according to procedures described by Iglesias *et al.* [7]. Naringin was dissolved in dimethyl sulphoxide (DMSO), and its concentration during incubation with sPLA2 never exceeded 1%. Purified sPLA2 (1.5 mg, 100 nmol/mL) was dissolved in 1 mL of water. After complete homogenization, 10 µL of naringin solution (100 nmol) was added and incubated for 60 minutes in a water bath at 37 °C. Samples (200 µL) were loaded onto a preparative reverse phase HPLC column to separate the modified sPLA2 (sPLA2-Nar) from naringin. Samples were eluted using a discontinuous gradient of buffer (66.6% acetonitrile in 0.1% TFA) at a constant flow rate of 2.0 mL/min. The chromatographic run was monitored at A280 nm. In addition to the chemical modification with naringin, another modification with p-BPB was carried out according to the protocol described by de Castro *et al.* [43] and Hernandez-Oliveira *et al.* [44].

### 3.4. Measurement of sPLA2 oxidation

Oxidation of sPLA2 after incubation with naringin was determined by measuring the absorption spectra of sPLA2 and sPLA2-Nar using wavelengths ranging from 200 to 300 nm. The chromatographic run was performed utilizing a Waters HPLC, with a 991 photodiode array detector (PDA 991, Waters) to record the absorption spectra of the samples. Detection was carried out at A214 nm, with peak scanning between 190 and 310 nm (3-nm steps). In addition, chemical modification of the amino acid residues of sPLA2 in the presence of naringin was evaluated by amino acid analysis. For this analysis, approximately 1 nmol of purified protein (sPLA2 or sPLA2-Nar) was hydrolyzed with 6 N HCl (200 μL) in the presence of 10 μL of phenol. Amino acid hydrolysis was performed at 106 °C for 24 h. Then, excess HCl was removed, and the hydrolyzed amino acids were redried with an aqueous solution of ethanol:water:triethylamine, 2:2:1 by volume. Post-column derivatization was performed with an aqueous solution of phenylisothiocyanate (ethanol-water-triethylamine-phenylisothiocyanate, 7:1:1:1 by volume). Both samples and amino acid standards were derivatized using a PICO-TAG amino acid analyzer system. The analysis of the phenylthiohydantoin-amino acid was also carried out using a PICO-TAG amino acid analyzer (Waters).

### 3.5. Measurement of sPLA2 activity

sPLA2 activity was measured following protocols described by Rigden *et al.* [45] and modified by Toyama *et al.* [37] in 96-well plates, using 4-nitro-3-octanoyloxybenzoic acid (4N3OBA, BIOMOL, USA) as substrate. Enzyme activity, expressed as the initial velocity of the reaction (Vo) was calculated based on the increase in absorbance after 20 minutes. All assays were performed with absorbance at 425 nm using a SpectraMax 340 multiwell plate reader (Molecular Devices, Sunnyvale, CA). After addition of native or naringin-treated sPLA2 (20 μg), the reaction mixture was incubated for up to 40 minutes at 37 °C, and absorbance was read at 10-minute intervals.

### 3.6. Myotoxic activity

Creatine kinase (CK) was assayed using the CK-NAc kit (Laborlab). Native sPLA2, sPLA2-Nar, or sPLA2 treated with p-BPB (sPLA2-p-BPB) (1 µg/µL in 50 µL) was injected into the left *gastrocnemius* muscle of male Wistar rats (90–110 g; n = 6). Control rats received an equal volume of 0.15 M NaCl. After 3 hours, the rats were anesthetized, and blood was collected from the abdominal vena cava into tubes containing heparin as an anticoagulant. The plasma was stored at 4 °C for a maximum of 12 hours before assaying. The amount of CK was then determined using 4 µL plasma, which was incubated for 3 minutes at 37 °C with 1.0 mL of the reagent according to the kit protocol. Activity was expressed in U/L.

### 3.7. Neurotoxic effect assay 

Male chicks (4–8 days old) were killed with ether, and the *biventer cervicis* muscle was removed [46,47] and mounted under a resting tension of 1 g in a 4-mL organ bath containing aerated (95% O2 + 5% CO2) Krebs solution (pH 7.5, 37 °C) in mM: 118.7 NaCl, 4.7 KCl, 1.88 CaCl2, 1.17 KH2PO4, 1.17 MgSO4, 25.0 NaHCO3, and 11.65 glucose. A bipolar platinum ring electrode was placed around the tendon, which ran the length of the nerve trunk supplying the muscle. Indirect stimulation was applied with a Grass S4 stimulator (0.1 Hz, 0.2 msec, 3–4 mV). Muscle contractions and contractures were recorded by connecting the preparation to a force displacement transducer (Narco Biosystems Inc.) coupled to a Gould RS 3400 recorder. Contractures to exogenously applied acetylcholine (ACh, 55 or 110 µM for 60 seconds) and KCl (5 mM for 120–130 seconds) were obtained in the absence of nerve stimulation prior to the addition of modified and non-modified sPLA2 (10 µg/mL) at the end of the experiment. The preparations were allowed to stabilize for at least 20 minutes before the addition of ACh or KCl and a single concentration (10 µg/mL) of the compounds.

### 3.8. Platelet aggregation studies

Platelet aggregation activity was measured following methods described by Fonseca *et al.* [48]. Venous blood was collected following informed consent from healthy volunteers who denied taking any medication in the previous 14 days. Blood was collected with a two-syringe technique using polypropylene syringes and 19-gauge needles, and was immediately transferred into polypropylene tubes containing one-tenth final volume of 3.8% trisodium citrate. After removing the platelet-rich plasma, the remaining blood was prepared by centrifugation of citrated blood at 200 g for 10 min. Washed platelets were obtained from the residue by centrifugation of citrated blood at 1,500 g for 20 min. Platelets were incubated for 1 hour at room temperature to recover their sensitivity to aggregating agents. Platelet counts were performed on a Coulter S Plus apparatus (Coulter Electronics, Hialeah, FL) or with phase-contrast microscopy. Platelet aggregation was carried out using 400 μL of the washed platelets solution in a cuvette and incubated at 37 °C with constant stirring.

The desired concentration of protein was added 3 minutes prior to the addition of platelet aggregation inducers (thrombin for washed platelets). Subsequently, the aggregation was recorded for 5–10 minutes with an aggregometer (Payton Scientific Instruments, Inc, Buffalo, NY). Aggregation experiments were performed with sPLA2 or sPLA2-Nar in concentrations of 10 μg in a final volume of 20 μL.

### 3.9. Paw edema assay

Male Wistar rats (120–150 g) were anaesthetized with halothane (inhaled). Hind paw edema was induced by a single subplantar injection of native sPLA2 or sPLA2-Nar (10 μg per paw). Paw volume was measured immediately before the injection of the drugs and at selected times thereafter (30, 60, 90, 120, 180, and 240 minutes) using a hydroplethysmometer (model 7150, Ugo Basile, Italy). All drugs were dissolved in sterile saline solution (0.9%). Results were expressed as the increase in paw volume (mL) calculated by subtracting the basal volume. When desired, the area under the time–course curve (AUC) was also calculated (trapezoidal rule), and the results were expressed as the total edema volume (mL per paw).

Thus, three groups of rats (n = 4 per group) were injected (i.p.) with dexamethasone or naringin (3.0 mg/kg) 30 minutes before the injection of native sPLA2 (10 μg per paw). Each rat received 0.1 mL of drug. Dexamethasone and PBS were used as controls to evaluate the anti-inflammatory effect of naringin. 

### 3.10. Antibacterial activity 

The antibacterial activity was assayed as described by Santi-Gadelha *et al.* [49], and structural modification was done according to protocols described by Toyama *et al.* [50]. *Xanthomonas axonopodis pv. passiflorae* (Gram negative) and *Streptococcus mutans* (Gram positive) cells were harvested from fresh agar plates and suspended in sterile distilled water (A600 nm = 3 × 108 CFU/mL). Aliquots of bacterial suspension were diluted to 103 colony-forming units/mL (CFU/mL) and incubated with sPLA2 and sPLA2-Nar samples (75 μg/mL) for 60 minutes at 28 °C. Survival was assayed on nutrient agar (Difco) plates (n = 5). For both antibacterial assays, electron microscopic assessment of morphologic alterations was performed in the absence (control) or presence of sPLA2 or sPLA2-Nar.

Bacterial samples were centrifuged, recovered, fixed with 4% glutaraldehyde in buffer A (0.1 M cacodylate buffer, pH 7.4) for 1 h at 4 °C, embedded in 3% low-melting temperature agarose (Sea Plaque, FMC Corp.), and stored for 12 h in fixative at 4 °C. Agarose pellets were immersed in fixative for 2 h at 25 °C, washed three times for 10 minutes with buffer A, and fixed with 1% osmium tetraoxide for 2 hours at 25 °C. Sections were washed three times, dehydrated in increasing concentrations of ethanol, and embedded in Epon resin. Polymerization occurred at 60 °C for 48 hours, and ultra-thin sections were prepared with a Sorvall MT2 ultramicrotome. Sections were placed on grids and stained with 2% uranyl acetate for 15 minutes, followed by 2.6% lead citrate for 15 minutes. Samples were observed under a LEO 906 transmission electron microscope operating at 40–100 kV.

### 3.11. Circular dichroism (CD) spectroscopy

Native and sPLA2 modified with naringin were dissolved in phosphate buffer (10 mM, pH 7.4) to a final concentration of ~7 μM and ~4 μM for sPLA2 and sPLA2-Nar, respectively. Samples were transferred to quartz cuvettes with an optical path length of 1 mm. CD spectra between wavelengths 185–300 nm were measured with a JASCO 710 apparatus using a bandwidth of 1 nm and a response time of 2–4 seconds. Scans were collected for each sample, and all spectra were corrected by subtraction of buffer blanks. Comparison of spectra was performed after concentration and optical path length normalization in terms of molar CD.

### 3.12. Mass spectroscopy

The molecular mass of sPLA2 and sPLA2-Nar were determined by matrix-assisted laser desorption ionization-time-of-flight (MALDI-TOF) mass spectrometry using a Voyager-DE PRO MALDI-TOF mass spectrometer (Applied Biosystems). One microliter of samples (sPLA2 and sPLA2-Nar) in 0.1% TFA was mixed with 2 µL of the matrix a-cyano-4-hydroxycinnamic acid, 50% acetonitrile, and 0.1% TFA (v/v). The matrix was prepared with 30% acetonitrile and 0.1% (v/v) TFA. Ion masses were determined with an accelerating voltage of 25 kV, with the laser operated at 2,890 µJ/com2, with a delay of 300 nanoseconds, and in the linear analysis mode.

### 3.13. Small angle X-ray scattering (SAXS)

SAXS measurements were carried out at the D02A-SAXS2 beamline of the Brazilian Synchrotron Light Laboratory [51]. Samples were kept at 4 °C in the sample cell [52], and data acquisition was performed taking several 600-second frames for each sample, which allowed for the control of any possible radiation damage and improved statistical errors. Data were collected at a sample-to-detector distance of 985.7 mm using a MAR CCD detector (MAR Research) with X-rays at a wavelength of 1.488 Å, to cover q values ranging from 0.013 to 0.34 Å-1, where q, the moment transfer vector, is defined as q = 4πsinθ/λ. Different concentrations of sPLA2 were measured to assess aggregation [53,54] and interparticle interference effects. The best data sets were collected for samples of native sPLA2 and sPLA2-Nar at ~4 mg/mL and ~7 mg/mL, respectively.

Initial data treatment of the scattering intensities was performed using Fit2D software [55]. Data were reduced to a 1D scattering profile and normalized according to intensity and sample attenuation. The net scattered X-ray intensities for the proteins were determined by subtracting a normalized buffer blank from each protein data set. Different frames for each sample were inspected and averaged using the program PRIMUS [56]. Distance distribution functions, p(r), and radius of gyration, Rg, were evaluated by the indirect Fourier transform method included in the program GNOM [57].

The Rg value was also estimated using Guinier analysis [58] in which the ln of I(q) was plotted against q2 to yield a straight line whose slope is proportional to Rg. Additional analysis was performed by plotting Iq2 against q (Kratky plot), which allows the evaluation of the degree of protein globularization [59]. The shape of the Kratky plot is sensitive to the conformational state of the macromolecule in solution. Globularly folded macromolecules have bell-shaped curves, whereas for completely unfolded states, a plateau or a slightly increasing function in the larger q range is observed [60]. Estimation of the molecular weight of sPLA2 and sPLA2-Nar in solution was performed with a relatively simple method included in the “SAXS MoW” web tool (http://www.ifsc.usp.br/~saxs/saxsmow.html), which is based on the invariant Q calculated from the Kratky plot [61].

Low resolution envelopes were restored from the scattering curves using the ab initio procedure included in the program GASBOR [62]. In this program, dummy residue models were generated by a random-walk Cα chain and folded in a way that minimizes the discrepancy between the calculated scattering curve from the model and the experimental data. To increase the reliability of the results, final models were obtained with the program DAMAVER [63] using a spatial average of 50 independent models. To clarify the visualization, the average model was represented by a surface-calculated model using the program NCSMASK [64]. Superimpositions of crystallographic structures of several PLA2s found in the Protein Data Bank (PDB, www.rcsb.org) and the most probable SAXS models derived from DAMAVAVER were done using the program SUPCOMB [65]. Crystallographic sPLA2 models used in the interpretation of the low resolution models were chosen based on the smallest discrepancy (Chi) between the experimental scattering data and the scattering intensities computed for these models using the program CRYSOL [66]. In addition, rigid body modeling with the program SASREF [67] and flexible docking with the program package SITUS [68] were performed, aiming for the best interpretation of the SAXS envelopes.

### 3.14. Additional computational analysis

Automated comparative homology modeling using the SWISS-MODEL web server [69] (http://swissmodel.expasy.org//SWISS-MODEL.html) was employed to generate an atomic coordinate model for C. d. cascavella sPLA2. PSIPRED [70,71] was used for secondary structure prediction (http://bioinf.cs.ucl.ac.uk/psipred/). Protein assemblies and interfaces analyses were performed with the PISA web server [72] (http://www.ebi.ac.uk/msd-srv/prot_int/pistart.html).

### 3.15. Statistical analyses

Results are reported as the means ± S.E.M. of n experiments. The significance of differences between means was assessed by an analysis of variance, followed by a Dunnett’s test when several experimental groups were compared with the control group. The confidence limit for significance was 5%.

## 4. Conclusions 

According to our results, the enzymatic activity of native sPLA2 from *C. d. cascavella* was strongly reduced after treatment with naringin, almost to the same level as the potent sPLA2 enzymatic inhibitor p-BPB (Figure 2a). These findings suggest that naringin and p-BPB may inhibit sPLA2 activity in a different way. Crystallographic studies of sPLA2 from *Bothrops jararacussu* venom have shown that pBPB interacting with His-48 an important residue involved with the the catalytic triad, whereas our results suggest that naringin modified several aromatic residues in the hydrophobic channel [28]. 

Based on the chemical modification and CD experiments, naringin interacted predominantly in the sPLA2 molecular region with the highest concentration of aromatic amino acid residues (histidines and serines, shown in Figure 8), which are found at the bottom of the hydrophobic channel (between the N-terminus and the α2- and α3-helices) and the β-wing motif. In addition to containing the majority of the modified amino acid residues, the hydrophobic channel is also the sPLA2 structural domain responsible for catalysis, comprising the catalytic triad: Gly31, His47, and Asp89 according to the sPLA2 amino acid sequence (shown in Figure 8). Thus, chemical and structural modifications in this enzymatic region by naringin may be the main reason for the inhibition of enzymatic activity and the correlated edema formation and antibacterial functions of sPLA2 from *C. d. cascavella*.

The structural studies we performed permitted identification of the probable region in *C. d. cascavella* sPLA2 that was affected by naringin. This region seems to be responsible for some of the pharmacological properties of this enzyme. Chemical and structural modifications also interfered with the active site of sPLA2 and affected its possible enzymatic allosteric behavior.
